# Disconnectivity between Dorsal Raphe Nucleus and Posterior Cingulate Cortex in Later Life Depression

**DOI:** 10.3389/fnagi.2017.00236

**Published:** 2017-08-02

**Authors:** Toshikazu Ikuta, Koji Matsuo, Kenichiro Harada, Mami Nakashima, Teruyuki Hobara, Naoko Higuchi, Fumihiro Higuchi, Koji Otsuki, Tomohiko Shibata, Toshio Watanuki, Toshio Matsubara, Hirotaka Yamagata, Yoshifumi Watanabe

**Affiliations:** ^1^Department of Communication Sciences and Disorders, School of Applied Sciences, University of Mississippi University, MS, United States; ^2^Division of Neuropsychiatry, Department of Neuroscience, Graduate School of Medicine, Yamaguchi University Ube, Japan; ^3^Nagato-Ichinomiya Hospital Shimonoseki, Japan; ^4^Department of Psychiatry, Yamaguchi Grand Medical Center Hofu, Japan; ^5^Shinwaen Hospital Onoda, Japan; ^6^Health Administration Center, Yamaguchi University Organization for University Education Yamaguchi City, Japan

**Keywords:** depression, neuroimaging, geriatric psychiatry, magnetic resonance imaging, dorsal raphe nucleus

## Abstract

The dorsal raphe nucleus (DRN) has been repeatedly implicated as having a significant relationship with depression, along with its serotoninergic innervation. However, functional connectivity of the DRN in depression is not well understood. The current study aimed to isolate functional connectivity of the DRN distinct in later life depression (LLD) compared to a healthy age-matched population. Resting state functional magnetic resonance imaging (rsfMRI) data from 95 participants (33 LLD and 62 healthy) were collected to examine functional connectivity from the DRN to the whole brain in voxel-wise fashion. The posterior cingulate cortex (PCC) bilaterally showed significantly smaller connectivity in the LLD group than the control group. The DRN to PCC connectivity did not show any association with the depressive status. The findings implicate that the LLD involves disruption of serotoninergic input to the PCC, which has been suggested to be a part of the reduced default mode network in depression.

## Introduction

Disruption of the dorsal raphe nucleus (DRN) is implicated in depression, given that the DRN serves as the major source of 5-HT release in the brain. In neuroanatomical studies, the area of the DRN is reduced in major depression (Matthews and Harrison, 2012). The number and density of neurons in this region is higher in suicide victims (Underwood et al., 1999). Physiologically, serotonin transporter dysfunction has been found in the DRN of patients with major depression (Hahn et al., 2014), and binding potentials for the 5-HT1A receptor have been found to be reduced in depression (Drevets et al., 1999). Polymorphisms in 5-HT1A gene have been found to be associated with antidepressants (Kato et al., 2009). Genetic variations of tryptophan hydroxylase 2 (*TPH2*) are also associated with major depression (Zill et al., 2004), and are further found to be associated with *TPH2* mRNA expression in the pons including the DRN (Lim et al., 2006). Single nucleotide polymorphisms of the 5-HT1A receptor and *TPH2* have been suggested to interact with the severity of depression and respond to SSRIs (Serretti et al., 2011; Jacobsen et al., 2012). Meanwhile, the DRN is understood to be critical for the actions of antidepressants including SSRIs and tricyclics (Briley and Moret, 1993), as well as for the pathophysiology of depression.

Forebrain 5-HT projections originate in the DRN (Törk, 1990; Baker et al., 1991). Rodent models further support disruption of the DRN in depression. Genetically introducing serotonin transporter deficiency in the DRN induces depression-like behavior in mice (Lira et al., 2003). Social stress has also been shown to influence serotoninergic neurons in the DRN of rats that adopted a proactive coping strategy against stress (Wood et al., 2013). More recently, the volume of the raphe nuclei, as well as other monoaminergic regions, have been shown to be negatively correlated with social avoidance, which indexes stress susceptibility (Anacker et al., 2016) The DRN cells have inhibitory projections to the cortex, including the cingulate gyrus (Olpe, 1981), which is also implicated in depression (Bae et al., 2006; Wise et al., 2016).

The pathophysiology of Later-Life Depression (LLD) is considered to be in parallel to the pathology of depression in younger ages, though LLD has certain defining characteristics, such as less genetic influence (Brodaty et al., 2001; Alexopoulos, 2005). Antidepressants have been shown to be especially effective with LLD (Salzman et al., 2002; Schneider et al., 2003). Therefore, it can be hypothesized that the role of the DRN is substantial in the pathology of LDD. The role of the DRN in LLD, however, is not clearly understood.

Neuropathological change of the raphe nuclei has been implicated in depression. Neuronal loss in the raphe nucleus of older patients with depression has been found in a postmortem study (Tsopelas et al., 2011). Lesser gray matter concentration of the DRN was found in patients with major depression compared to the healthy control group (Lee et al., 2011). Patients who had transient relapse of depression induced by tryptophan depletion showed greater correlation between habenula-DRN co-activity compared to those patients who did not show relapse (Morris et al., 1999). Recently, studies in the functional connectivity of the DRN in humans have shown positive connectivity to cortical regions, illuminating 5-HT associated regions (Beliveau et al., 2015). Yet, functional connectivity of the DRN in depression or LLD has not been extensively studied. Here, in order to elucidate the functional connectivity of the DRN, we examined the resting state of functional connectivity in LLD in comparison to an age-matched control cohort. We hypothesized that the disrupted DRN connectivity would be found in regions whose connectivity manifests a signature role of the DRN in neuropathophysiology of LLD.

## Materials and Methods

### Participants

We examined a total of 95 individuals, including 33 LLD patients and 62 healthy age-matched participants. All patients met the DSM-IV criteria for major depressive disorder. Mean age of the LLD group was 60.40 (SD = 7.82) years, and healthy group 62.73 (7.43) years. Patients were recruited from Yamaguchi University Hospital; referred by area clinics and hospitals; and diagnosed by clinical interviews performed by senior psychiatrists, case conferences with psychiatrists, and structured interviews using the Mini International Neuropsychiatric Interview (MINI, Japanese version 5.0.0; Otsubo et al., 2005). Patients’ clinical demographics were obtained through a clinical interview. Patients with current or a history of substance abuse/dependence or other psychotic illnesses were excluded from the study. Healthy control participants were recruited from the community through local advertisements and word of mouth. Any healthy subject who had psychiatric illness was excluded through the MINI and clinical interviews. Participants with an immediate family member with a psychiatric disorder were also excluded. Exclusion criteria included ambidextrous or left handedness (Oldfield, 1971), MR imaging contraindications, presence of serious medical conditions, and hospitalization in the prior 6 months. Current mood states were assessed using the Structured Interview Guide for the Hamilton Depression Rating Scale (SIGH-D; Williams, 1988). Social functioning was assessed by the Global Assessment of Functioning scale (GAF; American Psychiatric Association, 2000). Participants with scores of 23 or below on the Mini-Mental State Examination were considered demented and excluded (Folstein et al., 1975). Through interviews, blood tests, and physical examinations, subjects with endocrinological disease, head trauma, neurological disease, a family history of hereditary neurological disorders, or other medical conditions (i.e., hypertension, diabetes, active liver disease, kidney problems, or respiratory problems) were also excluded from the study. LLD was defined as depression in patients over 50 years old at the time of study participation. The mean number of depressive episodes was 2.3 (1.8) in patients with LLD. Thirty patients were under medication at the time of study participation. Five patients were treated only with antidepressants; eight with antidepressants and first-generation antipsychotics; 12 with antidepressants and second-generation antipsychotics; two with an antidepressant and a mood stabilizer; with antidepressant, a second-generation antipsychotic, and a mood stabilizer; one with a second-generation antipsychotic; and one was with a mood stabilizer. Among the remaining three patients, one was drug-naïve, and the other two had been medication-free at least for the previous 4 years. The mean imipramine-equivalent dose among all the patients was 174.5 (151.3) mg. Most patients were under one or more antidepressant medications: SSRI-16; SNRI-14; Tricyclics-8; Others (mirtazapine, trazodone and mianserin)-13. This study was conducted in accordance with the latest version of the Declaration of Helsinki. This study was approved by the Yamaguchi University Institutional Review Board and written informed consent was obtained from all study participants.

### Clinical Assessments

Current mood states were examined using the SIGH-D (Williams, 1988). To assess social functioning, the GAF (American Psychiatric Association, 2000) was used. Self-reported depression status was measured by the Beck Depression Inventory (BDI; Beck et al., 1961). To uniformly assess various antidepressant dosage Imipramine Equivalent Potency was calculated.

### MRI Data Acquisition

A total of 204 echo-planner imaging (EPI) volumes were acquired on a Siemens Magnetom Skyra system (TR = 2500 ms, TE = 30 ms, matrix = 64 * 64, FOV = 220 mm, slice thickness = 4 mm, 34 continuous axial slices) as well as the MPRAGE structural volume (TR = 2300 ms, TE = 2.95 ms, flip angle = 9°, matrix = 256 × 256, FOV = 270 mm, 1.2 mm thick sagittal images).

### MRI Data Processing

Script package from the 1000 Functional Connectomes Project^1^ (Biswal et al., 2010) was used for preprocessing and seed-voxel correlation matrix production whereby FSL^2^ and AFNI^3^ are called. Motion correction and spatial smoothing (6-mm FWHM Gaussian kernel) were carried out through preprocessing. Each individual rs-fMRI series was registered and normalized to MNI152 2 mm space via T1 volume. Through this registration, 12 affine parameters were created between rs-fMRI volume and MNI152 2 mm space, so that a seed ROI can be later registered to each individual rs-fMRI space. The rs-fMRI time series were band-pass filtered (between 0.005 Hz and 0.1 Hz). Each resting state volume was regressed by white matter and cerebrospinal fluid signal fluctuations as well as the six motion parameters.

The DRN seed was defined as a 32 mm^3^ region centered at [MNI: 0, −27, −9] following the literature (Kranz et al., 2012; Beliveau et al., 2015). From the DRN seed, voxel-wise connectivity analyses were conducted by the “singlesubjectRSFC fcon” script, whereby the time course is spatially averaged within the ROI so that correlations from the ROI to each individual voxel across the brain.

To compare the two groups, a Z statistic image was estimated where clusters were determined by a family-wise, error-corrected cluster significance threshold of *p* < 0.01 assuming a Gaussian random field for the Z-statistics.

In order to examine the association between the functional connectivity differences and status of depression, clusters that showed significant effect of group were tested for further association with depression scales within the LLD group. The mean Z-scores of a cluster were calculated and correlations between the mean score and each of the depression scales was tested.

## Results

The LLD group showed significant indications of depression; BDI (control: 7.15 ± 4.71; LDD: 29.53 ± 11.45), GAF (control: 93.02 ± 4.72; LDD: 52.79 ± 6.19), and SIGH-D 17 (control: 0.81 ± 1.07; LDD: 21.87 ± 3.49), as well as their antidepressant medications assessed as imipramine equivalent potency (control: 0 ± 0; LDD: 193.64 ± 145.75).

**Figure 1:**
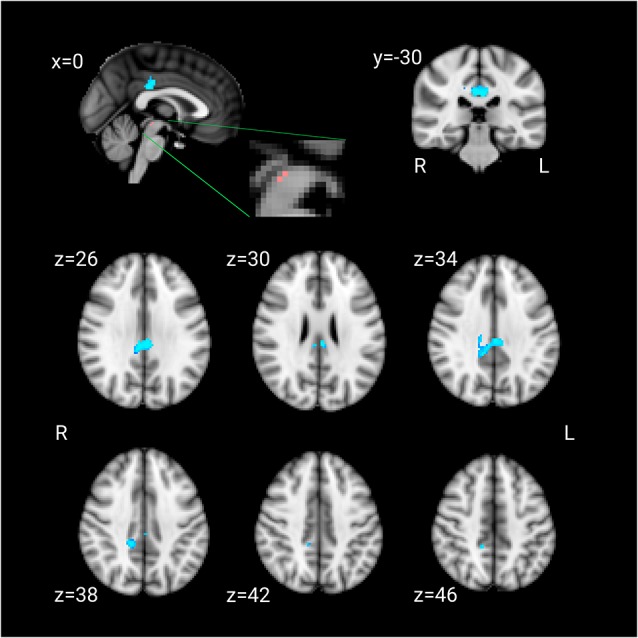
*Pink*: the dorsal raphe nucleus (DRN) ROI. *Blue*: the cluster that showed lesser connectivity with the DRN ROI in patients compared to the control group.

The LLD group showed significantly lower functional connectivity between the DRN and bilateral posterior cingulate cortex (PCC). There was no other cluster that showed lower functional connectivity in the LLD group. The cluster had 301 voxels and the peak voxel (corrected *p* < 0.001) was at [MNI: −4 −30 26] (Figure 1). No region showed greater connectivity in the LLD group than the control group.

In the LLD group, none of these depression scales (SIGH, BDI and GAF) or imipramine equivalent dose showed significant correlation to the functional connectivity between the DRN and cluster in the posterior cingulate cortices.

## Discussion

This study aimed to isolate the functional connectivity patterns of the DRN distinct in LLD. Here, the DRN showed deficient functional connectivity to the PCC in LLD. The results illuminate the serotoninergic dysfunction that may influence LLD pathology, which is independent from the depressive status.

The current results are solely based on the functional connectivity between two distant regions, and therefore does not grant anatomical connections between two regions. However, serotoninergic projections from the DRN to PCC has been well-documented in rodents (Olpe, 1981; Finch et al., 1984; Kosofsky and Molliver, 1987). In humans, depressive symptoms in Parkinson’s disease have been found to be associated with 5-HT transporter binding in the raphe nuclei and PCC, as well as other limbic structures (Politis et al., 2010), suggesting the association between depression and raphe-cingulate 5-HT projections. The current findings are consistent with raphe-cingulate serotoninergic projections that have been shown to be anatomically present.

Dysfunctions of the PCC have repeatedly been found in depression (Zhou et al., 2010; Berman et al., 2011; Leech and Sharp, 2014). At the same time, SSRIs have been shown to ameliorate PCC deficits. Sertraline and fluoxetine increase glucose metabolism in the PCC, correlating with clinical improvement (Buchsbaum and Hazlett, 1997; Mayberg et al., 2000). In addition, administration of SSRIs has been found to increase the PCC volume in the non-depressed, healthy population (Kraus et al., 2014). The current results are consistent with the previous PCC findings in depression. In the non-depressed, human population, the PCC has been shown to mediate emotion and memory related processes (Maddock et al., 2003), implicating its importance to mood disorders whereby impacts of emotional input are amplified. The PCC has been suggested to be associated with social/contextual self-reflection, duties or obligations, and autobiographical memory, suggesting PCC’s role in self rumination (Johnson et al., 2006, 2009; Svoboda et al., 2006; Herwig et al., 2012). The current results support the understanding that serotoninergic dysfunction at the DRN underlies LLD by showing the disruption of the connectivity between the DRN and PCC in LLD.

The PCC in non-depressed population has also been shown to play critical roles in the default mode network (Fox et al., 2005; Buckner et al., 2008; Fransson and Marrelec, 2008; Uddin et al., 2009). The PCC is one of the regions that is more active at rest than during a task (Buckner et al., 2008). Furthermore, the PCC activity negatively predicts the motor-control network (Uddin et al., 2009), implicating the importance of the PCC during rest. It may imply that the PCC may mediate cognitive process of *rest* through the default mode network. Remarkably, the default mode network has been found to be suppressed in major depressive disorder (Sheline et al., 2009).

The functional connectivity between the DRN and PCC was not significantly associated with any of depression scales or antidepressant dose, while LLD group showed significantly lesser connectivity compared to the control group. It may suggest that the reduced DRN-PCC functional connectivity is a trait characteristic of depression independent from the depressive state. The association between depressive traits and the PCC has previously been implicated. In a task fMRI study, greater activation of the PCC in depression was found during negative rumination tasks, compared to the healthy control group (Cooney et al., 2010). Increased dominance of the default mode network in depression, compared to the task positive network, was associated with higher levels of depressive rumination (Hamilton et al., 2011). Decreased connectivity of the PCC to the default mode network in depression has also been found in a resting state fMRI study and was associated with over general autobiographical memory (Zhu et al., 2012). Taken together, the PCC in depression is associated with negative rumination. Recall that the raphe serotoninergic projection to the PCC is inhibitory (Olpe, 1981). Our results can imply that decreased DRN-PCC connectivity reflect lesser inhibitory inputs to the PCC, which would downregulate negative rumination.

The imprecision of the DRN seed needs to be addressed as the limitation of this study. The DRN seed was defined in 2 mm^3^ space, which would not have sufficient resolutions to exclusively isolate the DRN. It should also be recalled that 6 mm smoothing was applied. Therefore, the seed may contain other structures around the DRN. Specifically, the median raphe nucleus (MRN) may not be excluded. In the previous study that differentiated resting state connectivity of DRN and MRN (Beliveau et al., 2015), [^11^C]DASB PET data were acquired in each subject and the DRN and MRN were localized in each individual brain. As we did not obtain PET data, we have far coarse localization of the DRN. The current finding might not be exclusively attributed to the DRN but it may also include the greater raphe nuclei including MRN. It would be ideal to conduct a similar study with PET data for more precise localization of the DRN.

The MRN also possesses serotoninergic characteristics which implicates an association with depression (Bach-Mizrachi et al., 2008). MRN has also been shown to modulate emotional behaviors (López Hill et al., 2013). The association with the hippocampus has been found more in the MRN than DRN (Jacobs et al., 1974). Also, the MRN has known efferent to the cingulate cortex (Azmitia and Segal, 1978). It might be more appropriate to apprehend the finding as Raphe—PCC disconnectivity in LDD.

There are other limitations of this study that need to be addressed. The relationship between medication and functional connectivity is unclear. The findings may be the effect of medication as opposed to the effect of LLD. Studies examining a non-medicated population could better discriminate these possibilities. Though no association was found between the DRN-PCC connectivity and imipramine equivalent dose, it does not exclude the possibility for the influence of medication. In addition, the experience of being in an MRI scanner may have influenced the groups differently. LLD may trigger differential neuronal activation as a function of the experience.

In conclusion, this study found disconnectivity between the DRN and PCC in the LLD group that is consistent with serotoninergic dysfunction of the DRN in depression.

## Author Contributions

KM and YW conceived and designed the experiments. KH, MN, TH, NH, FH, KO, TS, TW, TM and HY performed the experiments. TI, KM and KH analyzed the data and wrote the article.

## Conflict of Interest Statement

TI has received speaker’s honoraria from Eli Lilly, Daiichi Sankyo, and Dainippon Sumitomo. KM has received research donations from GlaxoSmithKline and Ohtsuka Pharmaceutical. YW has received research donations from MSD, GlaxoSmithKline, Eli Lilly and Company, Yoshitomiyakuhin, Shinogi, Pfizer, Janssen Pharma, Meiji Seika Pharma, FujiFilm RI Pharma, Takeda Pharmaceutical, Astellas, Dainippon Sumitomo Pharma, and Ohtsuka Pharmaceutical.

## Footnotes

^1^http://www.nitrc.org/projects/fcon_1000

^2^http://www.fmrib.ox.ac.uk

^3^http://afni.nimh.nih.gov/afni
